# ALUMINUM-ACTIVATED MALATE TRANSPORTER 1 (ALMT1) partially acidifies the rhizosphere in Pi deficiency-induced inhibition of primary root growth

**DOI:** 10.1080/15592324.2025.2590763

**Published:** 2025-11-28

**Authors:** Zhen Wang, Mingzhe Xia, Rui Ma, Zai Zheng

**Affiliations:** a School of Agriculture, Forestry and Medicine, The Open University of China, Beijing, People's Republic of China; b State Key Laboratory of Plateau Ecology and Agriculture, Qinghai University, Xining, People's Republic of China; c College of Life and Environmental Sciences, Hangzhou Normal University, Hangzhou, People's Republic of China; d National Key Laboratory of Tropical Crop Breeding, Institute of Tropical Bioscience and Biotechnology & Sanya Research Institute, Chinese Academy of Tropical Agricultural Sciences, Haikou, People's Republic of China

**Keywords:** Pi deficiency, ALMT1, malate, rhizosphere acidification, primary root growth

## Abstract

The inhibition of primary root (PR) growth is a major developmental response of Arabidopsis (*Arabidopsis thaliana*) to phosphate (Pi) deficiency. Previously, our laboratory demonstrated that under Pi deficiency, a blue light-triggered malate-mediated photo-Fenton reaction and a canonical Fenton reaction in root apoplasts together form an Fe redox cycle, which results in Pi deficiency-induced inhibition of PR growth by continuously producing hydroxyl radicals (·OH). In this model, blue light, malate, Fe^2+^, Fe^3+^, H_2_O_2_, low pH, and low Pi are critical components, and the LPR1/LPR2 and STOP1-ALMT1 modules are key regulators that affect the occurrence and extent of these chemical reactions. However, whether the function of ALMT1 in the Pi deficiency-induced inhibition of PR growth relies on low pH in the rhizosphere and, conversely, whether ALMT1 is involved in regulating rhizosphere acidification remain elusive. Here, we show that low pH in the rhizosphere is required for malate-mediated inhibition of PR growth under Pi deficiency. Moreover, although not the principal factor, ALMT1 facilitates rhizosphere acidification under Pi deficiency. Our results shed new light on the function of ALMT1 and rhizosphere acidification under Pi deficiency.

## Introduction

Plants take up phosphorus (P), an essential macronutrient for plant growth, development, and metabolism, from soil in the form of inorganic phosphate (Pi), the availability of which is quite limited in soils.^1^ The growth of Arabidopsis primary root (PR) is inhibited when the plant is grown in Petri dishes under Pi deficiency.^2^ Over the past few years, researchers have found a strong association between the accumulation of iron (Fe) in root apoplasts and the inhibition of PR growth, with many regulatory factors involved in this process.^3-10^ For example, ALMT1, an aluminum (Al)-activated malate channel, exudes malate into the rhizosphere in response to Pi deficiency. Subsequently, malate-mediated Fe chelation and LPR1, a ferroxidase that oxidizes Fe^2+^ to Fe^3+^, trigger peroxidase-dependent cell wall stiffening. This process rapidly inhibits cell elongation in the transition zone.^5^

Our laboratory previously reported that Pi deficiency-induced inhibition of PR growth is caused by an Fe redox cycle composed of a blue light-triggered malate-mediated photo-Fenton reaction and a canonical Fenton reaction in root apoplasts. As a product of the Fe redox cycle, Fe^2+^ reacts with H_2_O_2_ to continuously produce hydroxyl radicals (·OH), which are detrimental to cell activity, thereby inhibiting PR growth.^11^ Recently, we reported that the inhibition of PR growth under Pi deficiency is determined by light illumination on roots but not shoots, and the blue light signaling pathway plays a minor role in this process.^12^ In our model, low pH plays an important role because a low pH of approximately 3.0 is generally required for the blue light-catalyzed photo-Fenton reaction, which converts Fe^3+^ to Fe^2+^. Although this reaction could be facilitated by the addition of malate at approximately pH 5.0, the amount of product Fe^2+^ dramatically decreases when the environmental pH is above 5.5 in in vitro experiments.^11^ However, whether low pH in the rhizosphere contributes to the function of ALMT1 in Pi deficiency-induced inhibition of PR growth and whether ALMT1 is involved in rhizosphere acidification remain unclear.

In this study, we evaluated the effects of pH on PR growth in the presence of malic acid and the acidification capacity of *almt1* and *ALMT1*-overexpressing (*ALMT1 OX*) lines under Pi deficiency. These results indicated that low pH in the rhizosphere is required for malate-mediated inhibition of PR growth under Pi deficiency, and conversely, ALMT1 plays a partial role in regulating rhizosphere acidification. Our study provides a new perspective for understanding the function of ALMT1 and rhizosphere acidification under Pi deficiency.

## Materials and methods

### Plant materials and growth conditions

The T-DNA insertion line of *ALMT1* (SALK_009629C, *almt1*) was obtained from the Arabidopsis Biological Resource Center (ABRC). *ALMT1 OX* lines were kind gifts from Dr. Thierry Desnos (CEA Cadarache, France).^5^

Plants were grown as previously described.^12^ Briefly, surface-sterilized seeds were stratified at 4 °C for 2 d and sown on Petri dishes that were vertically disposed in a growing room (16 h photoperiod; 22−24 °C; 100  µmol m^–2^ s^–1^ light intensity). The Pi-sufficient (+Pi) medium was half-strength MS medium with 1% (w/v) sucrose, 0.1% (w/v) MES, and 0.8% (w/v) agarose (Biowest Regular Agarose G-10). The KH_2_PO_4_ in the +Pi medium was replaced with K_2_SO_4_ in the Pi-deficient (–Pi) medium.

### Detection of rhizosphere acidification

Rhizosphere acidification activity was measured as described previously with slight modifications.^13^^,^^14^ Seedlings were grown on 1/2 MS +Pi medium for 4 d and then transferred to +Pi and −Pi medium for 2 d. A 600-μL aliquot of +Pi or −Pi medium, composed of 1% sucrose and 0.006% bromocresol purple and adjusted to pH 5.8, was added to 24-well plates. Twelve 6-d-old seedlings were pooled into one sample and transferred to the plates. The plates were incubated in growth chambers for 4 hours, after which 100 μL of the solution from each sample was transferred to a fresh 96-well plate. Rhizosphere acidification capacity was assessed via the absorption of medium before and after seedlings were transferred at 590 nm (ΔA_590_).

### Statistical analysis

One-way ANOVA, Student’s t-test, and Tukey’s multiple comparison were used to analyze the experimental data in GraphPad Prism software (version 10).

## Results and discussion

### Low pH in the rhizosphere is required for malate-mediated inhibition of primary root growth under Pi deficiency

**Figure 1.**
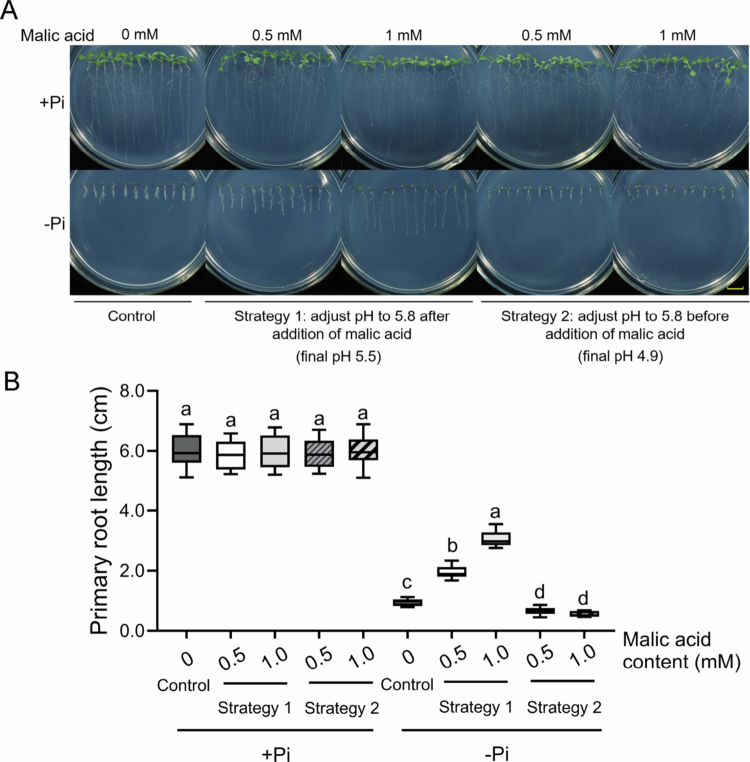
Influence of pH and malic acid application on the inhibition of primary root growth under –Pi conditions. The WT seeds were directly germinated on +Pi and –Pi media for 8 d, with 0.5 mM or 1 mM exogenous malic acid applied before (Strategy 1) or after (Strategy 2) adjusting the pH to 5.8. The final pH values of the media after autoclaving were 5.5 for Strategy 1 and 4.9 for Strategy 2. **(A)** Lengths of the primary roots of WT seedlings subjected to different concentrations of malic acid and different pH adjustment strategies. The bar = 1 cm. **(B)** Quantification of the primary root length of the seedlings in (A). The experiments were repeated three times, and representative results are shown. The boxplots contain the first and third quartiles, split by the median; Tukey whiskers point to the highest or lowest point. Different letters above any two columns within the same Pi condition indicate significant differences in values between these two samples (one-way ANOVA/Tukey test, *P* < 0.05).

Under Pi deficiency, malate exudation is crucial for Fe accumulation in the apoplast and subsequent peroxidase-dependent differentiation of meristematic cells.^5^^,^^7^ While exploring the function of malate in the Pi deficiency-induced inhibition of primary root (PR) growth, we made interesting observations. When we added malic acid to the −Pi medium first and then adjusted the pH value to 5.8, the inhibition of PR growth in the wild-type (WT) was abolished. In contrast, when we first adjusted the pH value to 5.8 and then added malic acid to the −Pi medium, the inhibition of PR growth was increased. Considering the varying pH values of these media, we further evaluated the impact of pH on PR growth under –Pi conditions with different concentrations of malic acid. The WT seeds were directly germinated on +Pi and –Pi media for 8 d, with 0.5 mM or 1 mM exogenous malic acid applied before (Strategy 1) or after (Strategy 2) adjusting the pH to 5.8. The final pH values of the media after autoclaving were 5.5 for Strategy 1 and 4.9 for Strategy 2. Under +Pi conditions, neither the application of malic acid nor the pH adjustment strategy had a significant effect on PR growth compared to seedlings without treatment (Figure 1). In the –Pi medium, when we employed Strategy 1 for pH adjustment, we observed that the application of exogenous malic acid did not significantly inhibit PR growth; moreover, PR growth appeared to be more tolerant to –Pi conditions. In contrast, when Strategy 2 was used for pH adjustment, the seedlings displayed hypersensitive PR growth compared with those without malic acid application (Figure 1). These results indicated that under Pi deficiency, a low pH in the rhizosphere is essential for the malate-mediated inhibition of PR growth.

In line with previous research, it has been suggested that rhizosphere acidification is necessary for the Pi deficiency-induced inhibition of PR growth. For instance, Svistoonoff et al. demonstrated that root growth arrest does not occur under −Pi conditions at a pH greater than 6.5.^3^ Balzergue et al. proposed that at a high pH of 7.1, *ALMT1 OX* does not lead to shorter roots under –Pi conditions.^5^ Godon et al. reported that the accumulation of STOP1 in the nucleus only occurs under acidic conditions (pH < 6.1) and in the presence of Fe.^15^ Therefore, we can infer that a low pH in the rhizosphere under –Pi conditions promotes the transcription of *ALMT1* via the nuclear accumulation of STOP1 and enables malate-mediated inhibition of PR growth under Pi deficiency.

### ALMT1 partially modulates rhizosphere acidification under Pi deficiency by unknown mechanisms

Research over the past few decades has indicated that the function of ALMT1 is closely associated with low pH in the rhizosphere. On one hand, the *ALMT1* gene is transcriptionally upregulated by low pH;^16^ on the other hand, when plants are exposed to aluminum (Al) toxicity, Al binds to the extracellular side of the ALMT1 channel and induces the opening of the extracellular gate at pH 5.0.^17^ Above, we found that the function of ALMT1 in the Pi deficiency-induced inhibition of PR growth also relies on low pH in the rhizosphere. Thus, we next investigated whether ALMT1 conversely plays a role in rhizosphere acidification under –Pi conditions.

**Figure 2.**
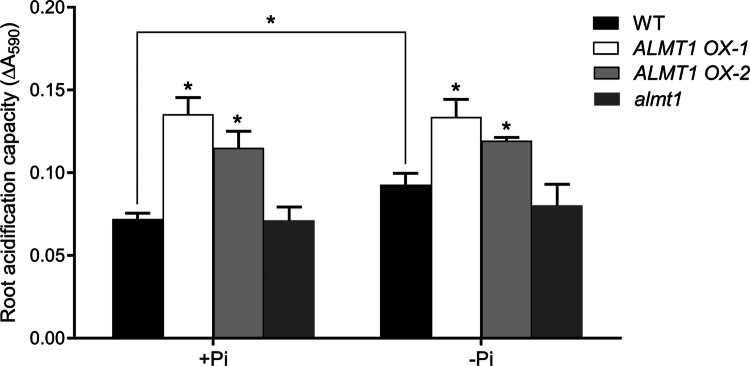
Rhizosphere acidification capacity of the *almt1* and *ALMT OX* lines under Pi deficiency. The seeds of the WT, *ALMT1 OX* lines, and *almt1* lines were grown on +Pi media for 4 d and then transferred to +Pi and −Pi media for 2 d. The rhizosphere acidification capacity was detected by rhizosphere acidification method and represented by adsorption at 590 nm (ΔA590). These experiments were repeated three times with similar results. The values represent means ± SD of more than ten primary roots for each genotype. Asterisks indicate statistically significant differences compared with those of the WT (Student’s t-test, *P < 0.05).

To this end, we directly measured the root acidification capacity of *ALMT1*-overexpressing (*ALMT OX-1* and *OX-2*) lines and the *almt1* mutant. The WT, *ALMT1 OX* seedlings, and *almt1* mutants were grown on 1/2 MS media for 4 d and then transferred to +Pi and −Pi media for 2 d before measuring their rhizosphere acidification capacity. We found that the WT seedlings exhibited significant acidification on –Pi medium compared with +Pi medium, while the degree of root acidification decreased in the *almt1* mutant and significantly greater in the *ALMT1 OX* lines under both +Pi and –Pi conditions (Figure 2). These results suggested that although ALMT1 is not the primary contributor, ALMT1 promotes rhizosphere acidification under –Pi conditions.

**Figure 3.**
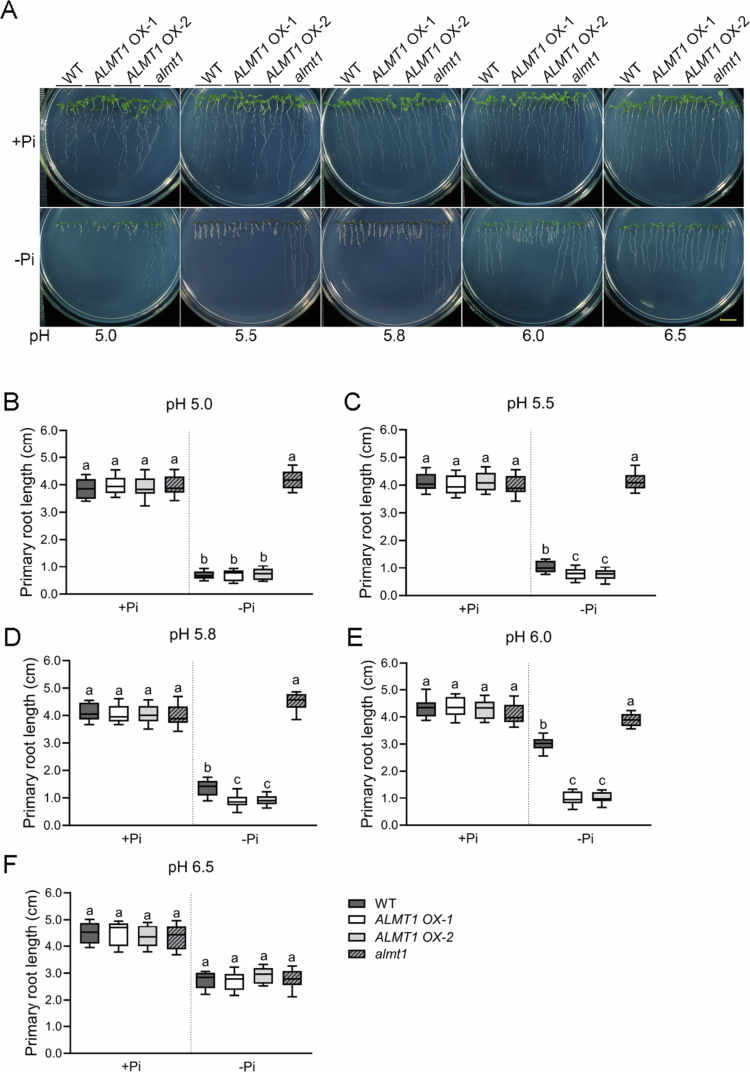
The primary root growth of *ALMT1 OX* lines is more resistant to environmental alkalization. The WT, *ALMT1 OX*, and *almt1* lines were grown on +Pi and −Pi media for 8 d under different pH conditions ranging from 5.0 to 6.5. **(A)** Lengths of the primary roots of the WT, *ALMT1 OX* lines, and *almt1* lines at different pH values. The bar = 1 cm. **(B)–(F)** Quantification of the primary root length of the seedlings in (A). The experiments were repeated three times, and representative results are shown. The boxplots contain the first and third quartiles, split by the median; Tukey whiskers point to the highest or lowest point. Different letters above any two columns within the same Pi condition indicate significant differences in values between these two samples (one-way ANOVA/Tukey test, *P* < 0.05).

On the contrary, if ALMT1 could increase rhizosphere acidification, the PR growth of *ALMT1 OX* lines should be more resistant to environmental alkalization. To test this hypothesis, the WT, *ALMT1 OX* lines, and *almt1* mutants were grown on +Pi and −Pi media for 8 d under different pH conditions ranging from 5.0 to 6.5. As the pH of the medium increased, the inhibition of PR growth in the WT and *ALMT1 OX* lines under −Pi conditions was gradually alleviated (Figure 3A–C), which is consistent with previous studies.^3^^,^^5^^,^^15^ However, at pH 6.0, the *ALMT1 OX* lines largely maintained the inhibition of PR growth under –Pi conditions, while the PR length of the WT approximately doubled relative to that at pH 5.8 (Figure 3D, E), suggesting that *ALMT1 OX* lines were more resistant to environmental alkalization.

**Figure 4.**
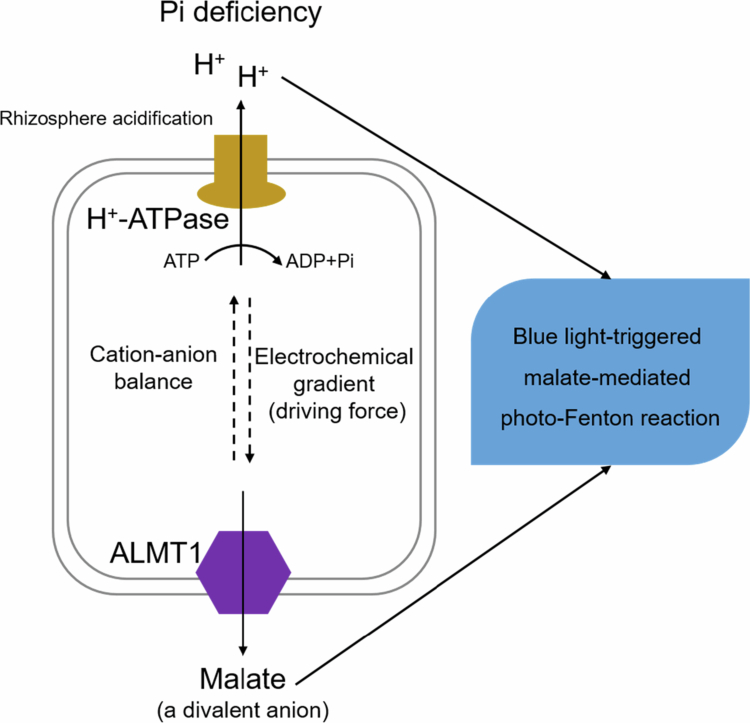
ALMT1 partially regulates rhizosphere acidification in −Pi roots by unknown mechanisms. Under Pi deficiency, the transcription and activity of H^+^-ATPases are upregulated, and many protons are pumped into the apoplast, resulting in rhizosphere acidification. Meanwhile, the transcription of *ALMT1* is upregulated, and a large amount of malate is exuded into the apoplast down the electrochemical gradient created by H^+^-ATPases. We assumed that the exudation of malate conversely caused the upregulation of H^+^-ATPases by cation–anion balance. Ultimately, the exuded malate and protons are involved in the blue light-triggered malate-mediated photo-Fenton reaction, which, in turn, causes Pi deficiency-induced inhibition of PR growth. The dashed lines indicate our proposed mechanism, which needs to be verified in the future.

Plants acidify the rhizosphere to promote the release of Pi from soil minerals. Previous studies have suggested that rhizosphere acidification under −Pi conditions is mainly a result of H^+^-ATPase-mediated proton efflux.^14^^,^^18^^,^^19^ The correlation between H^+^-ATPase activity and organic acid (OA) anion exudation in response to Al toxicity and Pi deficiency has been intensively investigated in various species.^19-21^ The H^+^-ATPase-induced electrochemical potential across the plasma membrane promotes the activity of organic acid transporters and a passive efflux of organic anions from the root tips.^19^^,^^22^ Therefore, we speculated that in Arabidopsis, the overexpression of the *ALMT1* gene results in a large amount of malate (a divalent anion) exudation. In this case, protons might be effluxed by H^+^-ATPases as counterions to compensate for the unbalanced cation-anion uptake at the soil-root surface, which additionally contributes to rhizosphere acidification (Figure 4). However, whether and how H^+^-ATPases are associated with this process remain to be investigated in the future. In transparent Patri dishes, the exuded malate and protons are ultimately involved in the Fe redox cycle, which leads to Pi deficiency-induced inhibition of PR growth, encouraging us to carefully evaluate the phenotype under illuminated, transparent Petri dishes.

## Conclusion

Previous work has shown that ALMT1, a malate channel localized to the plasma membrane, regulates the Pi deficiency-induced inhibition of PR growth by releasing malate into the apoplast of seedlings. In the present study, we demonstrated that the malate-mediated inhibition of PR growth under Pi deficiency requires a low pH in the rhizosphere. Further experiments revealed that ALMT1 partially contributes to rhizosphere acidification under –Pi conditions. ALMT1-mediated rhizosphere acidification might be caused by its collaboration with H^+^-ATPases via cation‒anion balance, which remains to be validated in future studies.
